# Gut microbiota and metabolic changes in children with idiopathic short stature

**DOI:** 10.1186/s12887-024-04944-3

**Published:** 2024-07-23

**Authors:** Luyan Yan, Bin Ye, Min Yang, Yongsheng Shan, Dan Yan, DanFeng Fang, Kaichuang Zhang, Yongguo Yu

**Affiliations:** 1 Department of Pediatric Endocrinology and Genetic Metabolism, Shanghai Institute for Pediatric Research, Xinhua Hospital Affiliated to Shanghai Jiao Tong University School of Medicine, Shanghai, China; 2 Department of Pediatric Internal Medicine, Taizhou Central Hospital, Taizhou University Hospital, Taizhou, China; 3 Department of Pediatrics, Shengjing Hospital of China Medical University, Shenyang, China; 4 Department of Pediatrics, Xiaoshan Hospital Affiliated to Hangzhou Normal University, Hangzhou, China

**Keywords:** Idiopathic short stature, Gut microbiome, Metabolomics, Metagenomic sequencing

## Abstract

**Background:**

Idiopathic short stature (ISS) is characterized by short stature with unknown causes. Recent studies showed different gut microbiota flora and reduced fecal short-chain fatty acids in ISS children. However, the roles of the microbiome and metabolites in the pathogenesis of ISS remains largely unknown.

**Methods:**

We recruited 51 Chinese subjects, comprising 26 ISS children and 25 normal-height control individuals. Untargeted metabolomics was performed to explore the fecal metabolic profiles between groups. A shotgun metagenomic sequencing approach was used to investigate the microbiome at the strains level. Mediation analyses were done to reveal correlations between the height standard deviation (SD) value, the gut microbiome and metabolites.

**Results:**

We detected marked differences in the composition of fecal metabolites in the ISS group, particularly a significant increase in erucic acid and a decrease in spermidine, adenosine and L-5-Hydroxytryptophan, when compared to those of controls. We further identified specific groups of bacterial strains to be associated with the different metabolic profile. Through mediation analysis, 50 linkages were established. KEGG pathway analysis of microbiota and metabolites indicated nutritional disturbances. 13 selected features were able to accurately distinguish the ISS children from the controls (AUC = 0.933 [95%CI, 79.9–100%]) by receiver operating characteristic (ROC) analysis.

**Conclusion:**

Our study suggests that the microbiome and the microbial-derived metabolites play certain roles in children’s growth. These findings provide a new research direction for better understanding the mechanism(s) underlying ISS.

**Supplementary Information:**

The online version contains supplementary material available at 10.1186/s12887-024-04944-3.

## Introduction

Short stature is defined as a height more than 2 Standard Deviation (SD) below the mean height for a given age, sex, and population. Idiopathic short stature (ISS) is the most common cause of short stature in children and excludes all possible causes such as undernourishment, endocrine conditions, skeletal dysplasia, genetic abnormalities and other systemic diseases [1]. At present, the etiology and pathogenesis of ISS remain unclear [2]. While recombinant human growth hormone (rhGH) therapy has been approved to treat children with ISS [3], the growth response to rhGH varies widely reflecting the heterogeneity of ISS [4]. Understanding the pathogenesis and disease mechanisms are important steps to identify new therapeutic targets and treatment.

Accumulating data has raised the possibility that gut microbiota may play an important role in body growth. Germ-free infant mice grew slower than the wild-type mice suggesting that the microbiota can interact with the somatotropic hormone axis to promote systemic growth [5]. In stunted undernourished children, a causal relationship was found between growth stunting and the components of the small intestinal microbiota [6]. Moreover, transplantation of immature microbiota from undernourished children into germ-free mice revealed that the immature microbiota impaired the growth phenotype [7]. Recently, a study using 16S rDNA sequencing reported that the composition of gut microbiota in ISS was very different from controls, with a significant decrease in the butyrate-producing genera including *Faecalibacterium* [8]. Targeted metabolomic analysis of fecal short-chain fatty acids from ISS children also revealed marked differences compared to healthy children [8].

Based on these pieces of evidence, we propose that the gut microbiota and resulting metabolic products may play an important role in the development of ISS. To test this hypothesis, we used shotgun metagenomic sequencing and untargeted metabolomics to analyze the gut flora in ISS and control individuals. We identified 50 associations between the height standard deviation (SD) value, microbiome and their derived metabolites by mediation analysis, which revealed that adenosine, erucic acid, spermidine, and L-5-Hydroxytryptophan could mediate the association of bacterial strains and the height SD value.

## Materials and methods

### Study design

From September 2019 to April 2021, we recruited 25 normal-height healthy children as controls from the general community and 26 children with ISS hospitalized in either the Xinhua Hospital Affiliated to Shanghai Jiao Tong University School of Medicine, Taizhou central Hospital, Shengjing Hospital of China Medical University or Zhejiang Xiaoshan Hospital. The study was approved by the Ethics Committee of Xinhua hospital (XHEC-C-2021–041-2), and has been registered in the Chinese Clinical Trial Registry (ChiCTR2100047810). Each hospital pediatric center strictly followed the same standard procedures for subject enrollment and for collection of clinical information and fecal samples. All subjects were of Hans Chinese ethnicity.

 The inclusion criteria for children with ISS were as follows: (1) Height more than 2 SD below the mean height for a given age, sex, and population; (2) Normal response to growth hormone during stimulation tests (> 10 ng/mL); (3) Normal body weight and length at birth; (4) Absence of all other possible causes of short stature including undernourishment, intrauterine growth retardation, endocrine conditions, skeletal dysplasia, chromosomal disease, inherited metabolic disease, mental illness and other chronic systemic diseases; (5) Normal or slow growth rate; (6) Normal or delayed bone age; (7) Age 3–14 years. The inclusion criteria for the controls were as follows: (1) Normal height for a given age, sex, and population; (2) Age 3–14 years. For all subjects, the exclusion criteria were as follows: (1) Autoimmune disorders such as inflammatory bowel disease, autoimmune thyroid disease and irritable bowel syndrome; (2) Gastrointestinal diseases such as cholelithiasis; (3) History of gastrointestinal surgeries such as gastrectomy, colectomy, ileectomy, cholecystectomy and appendectomy; (4) Neuropsychiatric disorders including autism, epilepsy and depression; (5) History of malignant tumors; (6) Other diseases including obesity, hypertension and diabetes mellitus; (7) Use of antibiotics, probiotics, hormones, proton pump inhibitors, insulin sensitizers or growth-promoting herbs in the previous three months; (8) Familial short stature. To reduce patient bias in the two groups, subjects were matched as closely as possible in terms of sex, age and living area by province.

### Fecal sample collection and DNA extraction

Written informed consent was obtained from all subjects meeting inclusion and exclusion criteria. Fresh stool samples were collected using disposable fecal collection tubes (Orienter, China), and immediately preserved at -80 °C until further analyses. Genomic DNA was extracted from fecal samples using Magnetic Soil And Stool DNA Kit (Qiagen, US) (Batch 1) or E.Z.N.A.® Soil DNA Kit (Omega Bio-tek, US) (Batch 2). DNA integrity was assessed by agarose gel electrophoresis and DNA concentrations measured using NanoDrop2000 (Thermo Fisher Scientific, US).

### Metagenomic sequencing and data analysis

#### Construction of sequencing libraries

Genomic DNA was fragmented to an average size of about 350 bp (Batch 1) or 400 bp (Batch 2) for library construction. The library was constructed using NEBNext® UltraTM DNA Library Prep Kit for Illumina (NEB, US) (Batch 1) or NEXTflexTM Rapid DNA-Seq (Bioo Scientific, US) (Batch 2). Adapters containing the full complement of sequencing primer hybridization sites were ligated to the blunt end of fragments.

#### Processing of sequencing data

Libraries were sequenced on the Illumina NovaSeq 6000 (Illumina, US). Raw sequencing reads were processed to acquire clean data using Trimmomatic v0.39 softwareand the following steps: (1) Removal of low-quality reads which contain more than 35 bp of ‘N’ bases (default quality threshold value ≤ 15); (2) Removal of reads which shared overlapping sequences above a certain portion with the adapter (default length of 10 bp).

Since the sequencing data for microbial DNA are polluted by host DNA and animal DNA derived from diet, a blast analysis of host databases using Bowtie2.4.1 software (http://bowtie-bio.sourceforge.net/bowtie2/index.shtml) was used to filter out contaminating reads, enriching for microbial sequences.

#### Metagenome assembly

High-quality paired-end reads from each sample were de novo assembled into at least 500bp scaffolds using SOAPdenovo software v2.04 (http://soap.genomics.org.cn/soapdenovo.html). Assembled scaffolds were interrupted from the N connections and scafftigs without N bases collected as a set for further analysis.

#### Gene prediction and abundance analysis

1. The ORF prediction for the assembled set of microbial scafftigs were performed using MetaGeneMark v2.10 software (http://topaz.gatech.edu/GeneMark/) and ORFs less than 100 nt were filtered out.
2. For ORF prediction, CD-HIT software v4.8.1 (http://www.bioinformatics.org/cd-hit) was used to remove the redundant sequences and obtain a non-redundant initial gene set (nucleic acid sequences encoded by non-redundant consecutive genes were called genes).
3. Genes in each sample were calculated by comparing the clean data to the initial gene set using SOAP2 software v2.21 (http://soap.genomics.org.cn/). The final gene set (Unigenes) for further analysis were obtained by filtering out the genes with less than two reads per sample.
4. The abundance of each gene in individual samples was then calculated by comparing the number of reads and considering gene length.
5. Batch normalization was performed using ComBat_seq (with batch and group information) from sva (R package).

#### Taxonomy annotation

1. Comparisons between the unigenes and bacterial, fungal, archaeabacteria and viral sequences extracted from NCBI NR databases (version 202,004, https://www.ncbi.nlm.nih.gov/) was performed using DIAMOND software v2.0.8.146 (https://github.com/bbuchfink/diamond/).
2. For sequence comparison, we selected those with an evalue less than the minimum evalue * ten. Because of the possibility of multiple outputs for each sequence, the Least Common Ancestors (LCA) algorithm (system classification applied to MEGAN, https://en.wikipedia.org/wiki/Lowest_common_ancestor) was used to determine taxonomy.
3. An abundance table with the number of genes for individual samples at each taxonomic rank (phyla, family, genus and species) was then generated. The abundance for a particular species in one sample was defined as the sum of gene abundance annotated as that species. The number of genes for a particular species in one sample was defined as the number of genes (not equal to zero) annotated as that species.

#### Functional database annotations

1. Using DIAMOND software v2.0.8.146 (https://github.com/bbuchfink/diamond/), we compared the unigenes with KEGG database version 2019.10 (http://www.kegg.jp/kegg/). For comparisons, we selected the best blast hits for further analysis.
2. From these comparisons, the number of genes for each sample at different taxonomic ranks was obtained. The number of genes for a particular function in different samples is equal to the number of genes functionally annotated whose abundance is not equal to zero.

#### Binning analysis

1. Software mmseq2 version 13.45111 (https://github.com/soedinglab/MMseqs2) was then applied to build unique scaftig collections. Next bowtie2 was used to build unique scaftig collection indexes and map the reads back to this collection.
2. Software metabat2 version 2.15 (https://bitbucket.org/berkeleylab/metabat) was used to run the binning process based on each sample scaftig depth data. A single binning process was first conducted using scaftigs depth data from all samples followed by a mixed binning process. Merged single binning and mix binning results were generated by dRep v3.2.0 (https://github.com/MrOlm/drep). CheckM v1.1.3 (https://github.com/Ecogenomics/CheckM) and finally used for bin genome completeness and contamination evaluation. Bins were retained with completeness > 75% and contamination < 10%.

### Untargeted metabolomics analysis

Fresh fecal tissues frozen in liquid nitrogen were ground and metabolites extracted with 80% methanol. The supernatant was injected into an LC–MS/MS system and analysis performed using the Vanquish UHPLC system (ThermoFisher, Germany) coupled with an Orbitrap Q ExactiveTM HF mass spectrometer (Thermo Fisher) in both positive and negative ionization modes(Some metabolites are more prone to adhering to hydrogen cations, resulting in the formation of molecular ion peaks easily in the positive mode, while others are more likely to lose hydrogen cations, facilitating the formation of molecular ion peaks in the negative mode). The raw data from each sample was then analyzed by Compound Discoverer 3.1 software (ThermoFisher) to obtain a list of peaks with retention time, m/z, and integrated peak area. The peak intensities were normalized and matched with a database to obtain accurate qualitative and quantitative measurements which were annotated using the KEGG (https://www.genome.jp/kegg/pathway.html), HMDB (https://hmdb.ca/metabolites), and LIPIDMaps (http://www.lipidmaps.org/) databases. After filling the gaps for missing values, different peak intensities of samples were then normalized to the total spectral intensity. Metabolite features with coefficient of variation (CV) ≥ 30% were then excluded after normalization.

### Statistical analysis

#### Untargeted metabolomics analysis

The differential metabolites were screened by Partial least squares discriminant analysis (PLS-DA) using R package ropes. Screening criteria for key metabolites were as follows: (1) Variable Importance in Projection** (**VIP) > 1; (2) The absolute value of the logarithm base 2 of the fold change was > 1. Metabolic pathways with P-values < 0.05 were considered as significantly enriched.

#### Distance matrix-based variance estimation

Applied feature selection based on the permutational multivariate analysis of variance (PERMANOVA) using euclidean distance was used to estimate the contributions of clinical factors to different omics data (metabolomics and microbiome).

#### Microbial diversity analysis

For α-diversity, microbiome diversity was evaluated by the Shannon index through R package vegan. Microbiome richness was evaluated by the number of features and microbiome evenness calculated by the Shannon index divided by the logarithm base 2 of richness. For analysis of the microbiome β-diversity, R package vegan was also used to perform principal coordinate analysis and PERMANOVA (Jaccard distance).

#### Linear discriminant analysis effect size (LEfSe)

LEfSe was used to determine the differential microbiota characteristics (strain level and KEGG modules). A Linear Discriminant Analysis (LDA**)** score > 2 in the LEfSe model was used as the screening criteria.

#### Associations with clinical parameters

Associations between clinical parameters and metabolomics and gut microbiome (strain level) were assessed by Spearman correlation and the false detection rate (FDR) calculated using the Benjamini–Hochberg procedure.

#### Prediction model

Random forest (RF) models were built using the R package RandomForest to distinguish key features associated with the ISS and control cohorts. Randomly, 70% of individuals were selected as a training dataset and 30% of individuals were selected as a testing dataset. Five repeats of tenfold cross-validation were then used to estimate model performance. A ROC curve and plot of the test model was generated using the R package pROC procedure.

#### Biomarkers identification through recursive feature elimination

We used a recursive feature(RF) elimination approach to identify the smallest subset of features that could produce an effective model with good prediction accuracy. In brief, this involved the iterative fitting of RF, whereupon each iteration, a specified proportion of variables, with the smallest variable importance progressively discarded. The R package Caret process was applied recursively until only a single variable remains available as input. At each iteration, the model performance is assessed in terms of the out-of-bag error, where RF is used in a classification capacity or mean squared error is used for regression forests. Following this sequence, a set of variables with the smallest number predictive features was identified.

#### Bi-directional mediation analysis

By Spearman correlation (FDR < 0.05), we first checked whether any clinical parameters were associated with the differential metabolites. Next, we carried out a bi-directional mediation analysis with interactions between mediator and outcome, using the mediation function from mediation software version 4.5.0 to infer the mediation effect of metabolites and microbiome with clinical parameters. The FDR was calculated based on the Benjamini–Hochberg procedure.

## Results

### Study groups

Based on strict inclusion and exclusion criteria, a total of 51 participants were enrolled comprising 26 subjects with ISS and 25 controls. Variables such as age and gender in the two groups were generally matched to reduce complications. The clinical characteristics of children with ISS and controls are summarized in Table 1 and Supplementary files. Spearman’s correlation analysis between clinical characteristics and the height SD value identified a significantly positive correlation with BMI (*p* < 0.05). For all 51 fecal samples analysed, we obtained meaningful data on gut microbiome and metabolic derivatives for statistical analysis.

**Table 1:** Characteristics of the study groups

Parameters	ISS(*n* = 26)	Control(*n* = 25)	*P* value
Gender(M/F)	13/13	13/12	0.886
Age(years, mean ± SD)	7.72 ± 3.19	7.90 ± 2.56	0.598
Height(cm, mean ± SD)	113.96 ± 16.30	128.61 ± 17.07	0.006
Height SD(mean ± SD)	-2.45 ± 0.46	0.14 ± 0.99	< 0.001
Weight(kg, mean ± SD)	19.56 ± 6.67	24.42 ± 8.00	0.002
BMI(mean ± SD)	14.70 ± 1.83	16.04 ± 2.12	0.008

The statistical difference in age between the two groups was analyzed using the chi-square test, while the statistical differences for other parameters were assessed with the Mann–Whitney U test

### Metabolic profiling of children with ISS

**Fig. 1:**
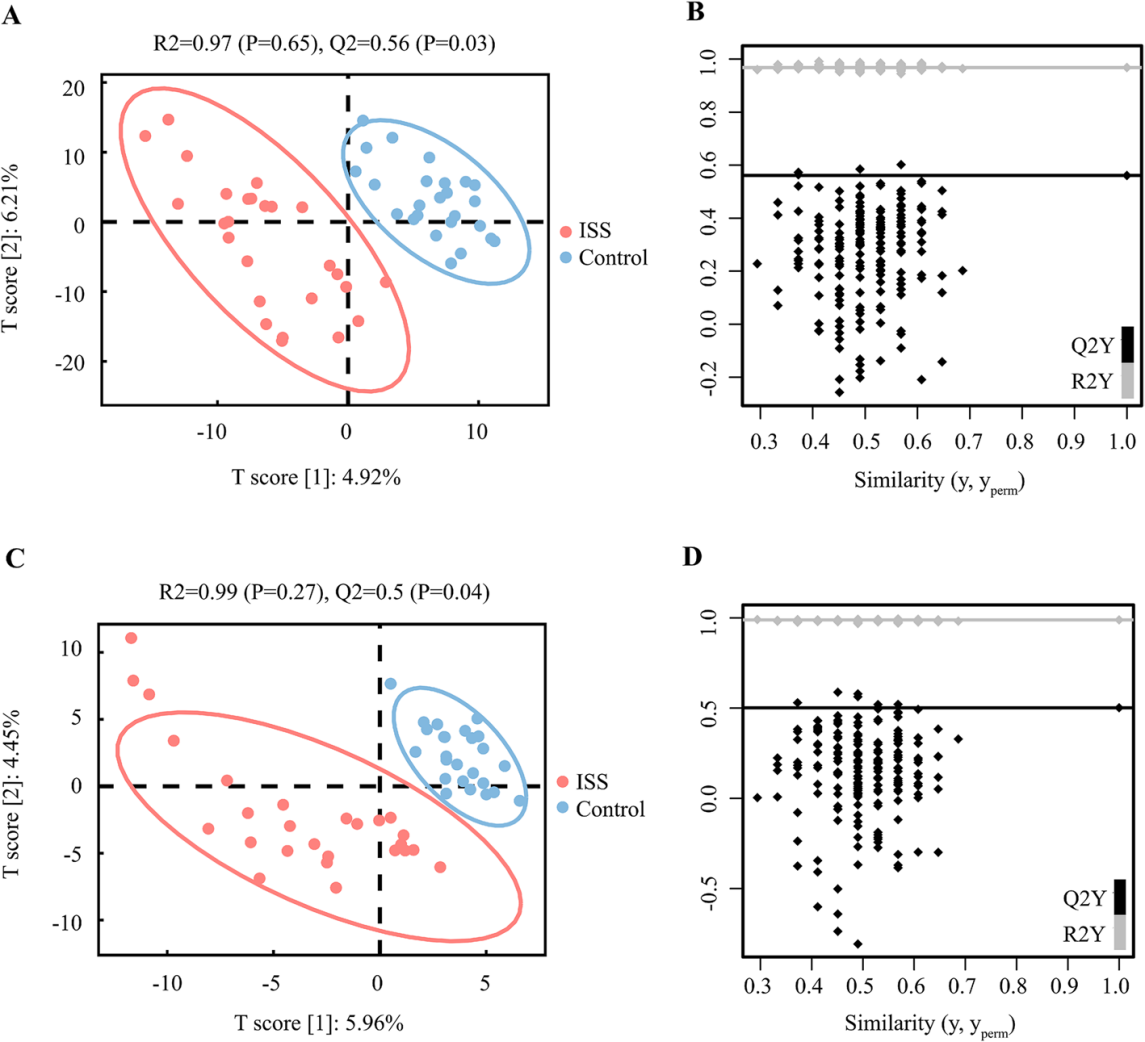
Differential metabolic profiles in individuals with ISS and Controls. **A** PLS-DA score plot in positive mode (R2 = 0.97, and Q2 = 0.56). **B** PLS-DA significance test in positive mode(pR2Y = 0.65, pQ2 = 0.03). **C** PLS-DA score plot in negative mode (R2 = 0.99, and Q2 = 0.5). **D** PLS-DA significance test in negative mode (pR2Y = 0.27, pQ2 = 0.04)

We used untargeted LC–MS to detect the composition of key fecal metabolome features. In total, 1987 compounds were observed in the positive and negative modes of LC–MS. PLS-DA score plots separated the ISS and control groups in both positive and negative modes, suggesting the presence of metabolic disturbances in the ISS children. Here, the R2 and Q2 scores were 0.97 and 0.56 in the positive mode (Fig. 1A) and 0.99 and 0.50 in the negative (Fig. 1C), respectively. The PERMANOVA test showed that the PLS-DA model was not overfitting and thus was valid for this study (positive mode: Fig. 1B, negative mode: Fig. 1D).

**Fig. 2:**
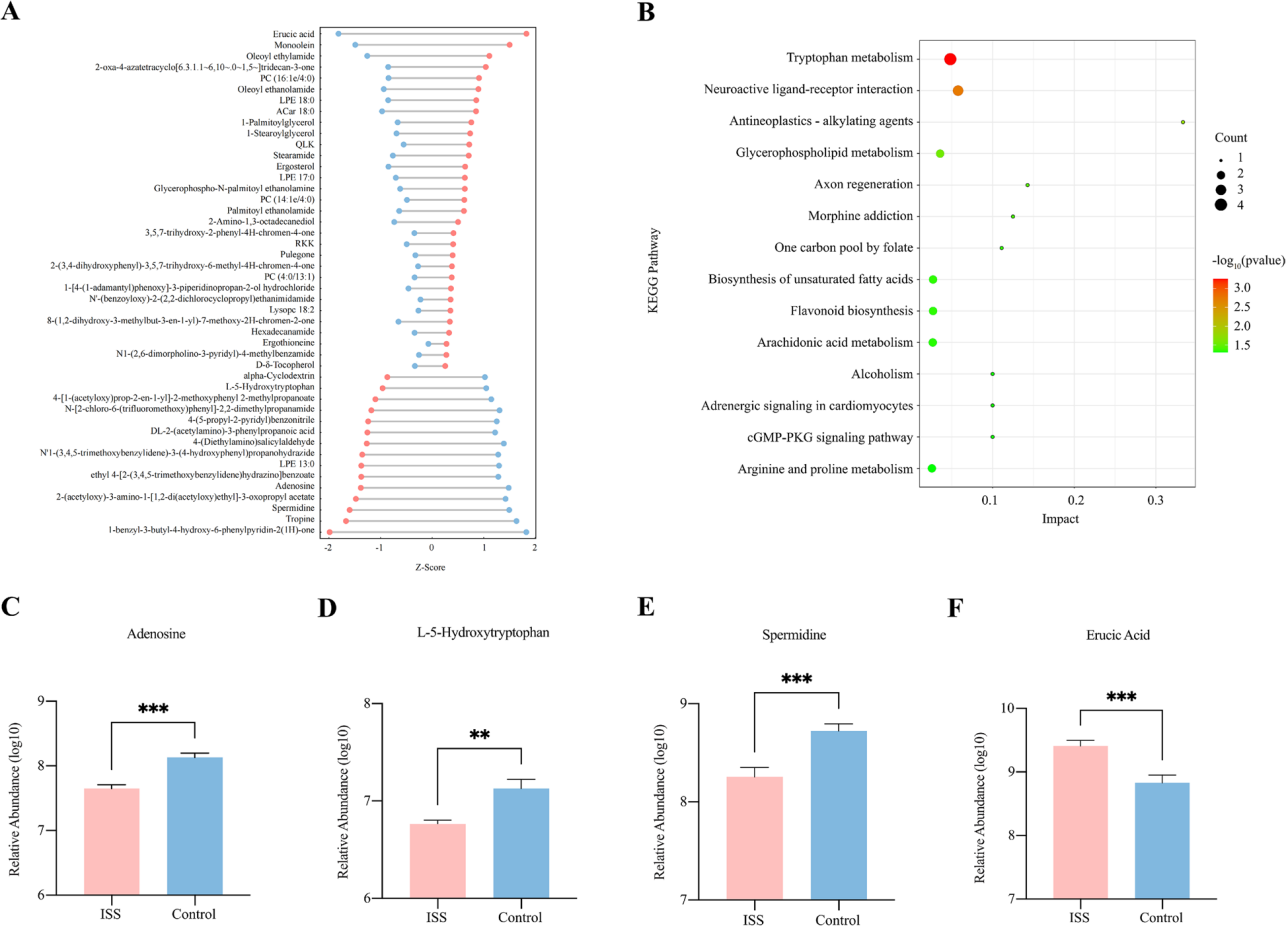
Identification of the fecal differential metabolites and pathways between the ISS and Control group in positive mode. **A** Visualization of differential metabolites. **B** The bubble plot displayed significantly enriched pathways (*p* < 0.05), **C** The relative intensity of the adenosine was significantly decreased in ISS. **D** The relative intensity of the L-5-Hydroxytryptophan was significantly decreased in ISS. **E** The relative intensity of the spermidine was significantly decreased in ISS. **F** The relative intensity of the erucic acid was significantly increased in ISS. Red and blue colours denote ISS and Control groups, respectively. ** *p*-value < 0.01; *** *p*-value < 0.001

Under the set cut-off conditions for the PLS-DA model (VIP > 1 and log2(FoldChange) > 1), we identified differential metabolite derivatives between the ISS and control groups in positive mode (Fig. 2A). Compared with controls, ISS exhibited an increase in organic acid, phosphatidyl choline, esters and fatty acids and, a decrease in amino acids, nucleotides, polyamines and lysophosphatidyl ethanolamine. A total of 52 metabolic pathways were enriched in the positive mode, among which 14 pathways exhibited a significant difference (*p* < 0.05, Fig. 2B). We observed that most of these pathways were concentrated in lipid and amino acid metabolism, such as tryptophan, glycerophospholipid, arginine and proline metabolism and, in the biosynthesis of unsaturated fatty acids and arachidonic acid metabolism. The altered metabolic profiles suggested a disturbance in nutrient metabolism in ISS children. Among these, four metabolites in particular had significant change, including increased level of erucic acid and decreased levels of spermidine, adenosine, and L-5-Hydroxytryptophan in ISS (Fig. 2C-F).

### Microbial community analysis

We investigated gut microbial alterations in ISS children using metagenomic sequencing, and the clean reads across the 51 samples ranged from 38835568 to 53845134 (44417337 ± 3355970, mean ± SD) after QC of the raw sequencing data. After correcting batch effects (Supplementary files), we performed data analysis. The microbial community compositions between the two groups were similar at the rank of phylum with *Firmicutes*, *Bacteroidetes*, *Actinobacteria*, and *Proteobacteria* being the most abundant phylum (Supplementary files).

**Fig. 3:**
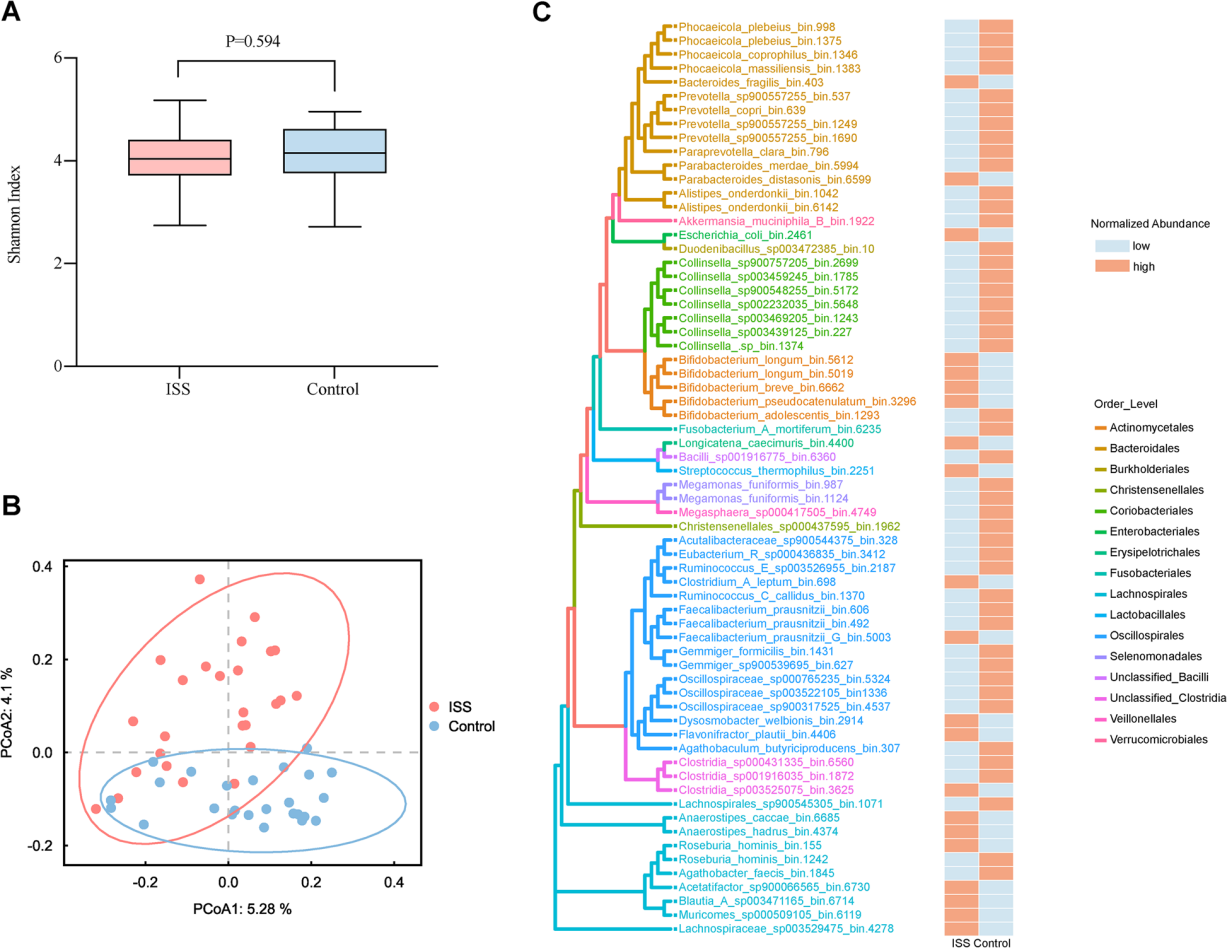
The alteration of the gut microbiota composition in individuals with ISS and Controls. **A** Shannon index showed no difference in α diversity (*p*_Shannon_ = 0.594). **B** Principal coordinate analysis (PCoA) analysis showed that the gut microbiota of individuals with ISS significantly differed from Controls (*p*_Adonis_ = 0.001). **C** Cladogram indicated the phylogenetic distribution of strains correlated with the ISS or Control groups

To identify any differences in bacterial diversity and richness between the two groups, we performed alpha diversity analysis, and initial results showed that the differences were not significant (*p*_Shannon_ = 0.594) (Fig. 3A). Next, we evaluated the beta diversity to compare the composition similarity between the microbiota communities and observed the gut microbiota composition. From this analysis, we found that ISS children were significantly different from controls (*p*_Adonis_ = 0.001, R2 = 0.033) (Fig. 3B). Comparing the relative percentages of bacterial genera between ISS and control children, we identified 7 genera with an increased abundance and 7 genera that had a decreased abundance in ISS children (Supplementary files). At the species level, the relative percentages of bacterial species in ISS were significantly different from those of controls, including 21 species with an increased abundance and 34 species with a decreased abundance (Supplementary files). We further evaluated microbial communities at the strain level. Using a total of 5309009 contigs across all 51 samples to bin the metagenome-assembled genomes (MAGs) and 333 high-quality MAGs (completeness > 75%; contamination < 5%) were generated. These MAGs were annotated to the database and taxonomy classifications at the strain level were obtained. A total of 66 differentially abundant microbial strains exhibited significant changes in abundance, of which 23 strains (33.3% were *Lachnospirales*, 19% were *Actinomycetes,* 19% were *Oscillospirales,* and 9.5% were *Bacteroidales*) were significantly increased in ISS and 43 strains (26.6% were *Bacteroidales,* 26.6% were *Oscillospirales,* 15.5% were *Coriobacteriales,* and 6.6% were *Lachnospirales*) were significantly decreased in ISS (Fig. 3C). Most MAGs were assigned to *Oscillospirales* (24.2%), followed by *Bacteroidales* (21.2%), *Lachnospirales* (15.2%), *Coriobacteriales* (10.6%) and *Actinomycetes* (7.6%).

**Fig. 4:**
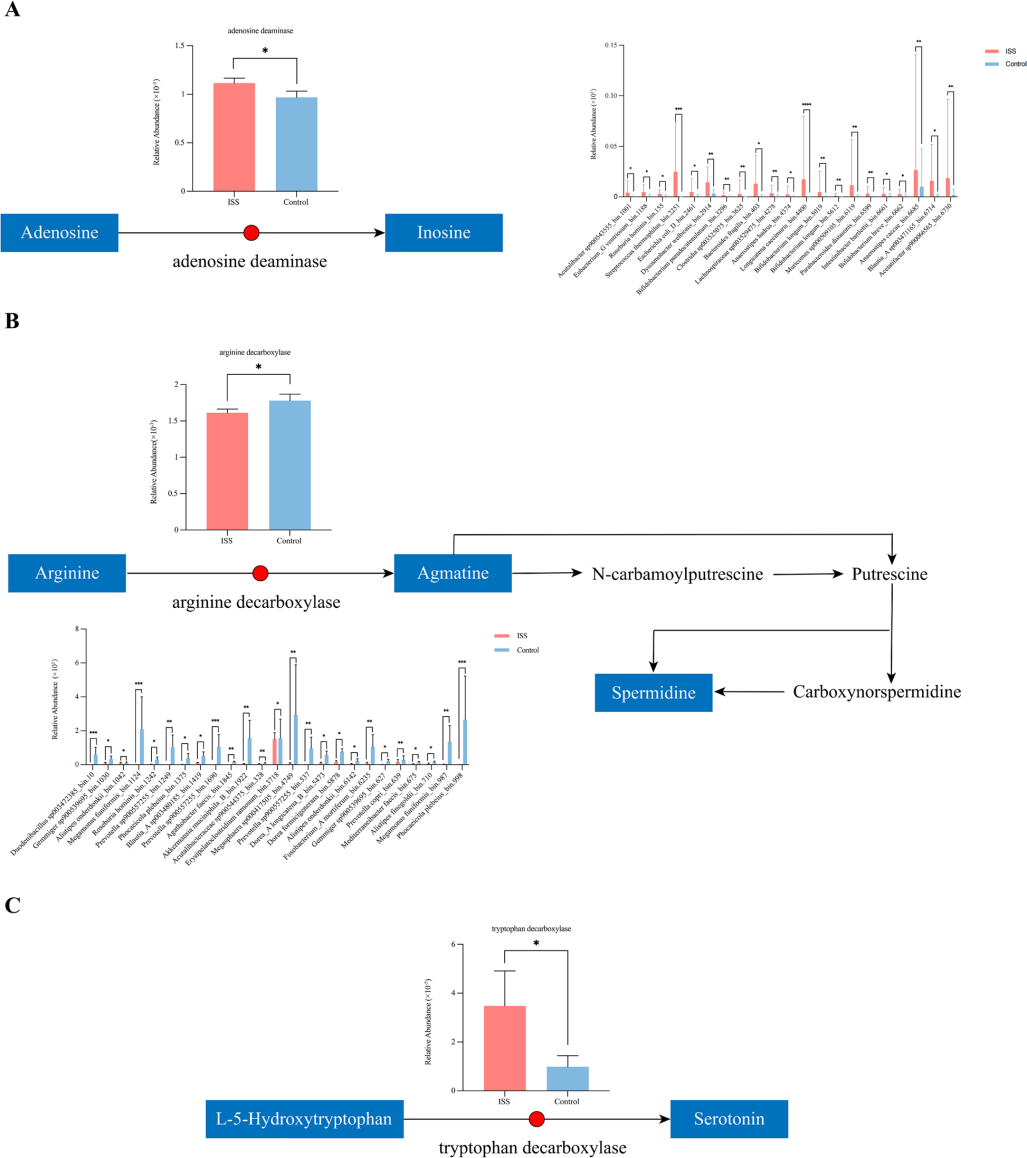
Key metabolic pathways mapped by microbial enzyme and fecal metabolism. **A** Microbial adenosine deaminase that converts adenosine to inosine was up-regulated in ISS. 21 bacterial strains that expressed adenosine deaminase were up-regulated in ISS. **B** Microbial arginine decarboxylase involved in the arginine metabolism was down-regulated in ISS. 25 bacterial strains containing arginine decarboxylase were down-regulated in ISS. **C** Microbial tryptophan decarboxylase involved in L-5-Hydroxytryptophan metabolism was up-regulated in ISS. * *p*-value < 0.05; ** *p*-value < 0.01; *** *p*-value < 0.001

A total of 31 differential KEGG modules were identified between the two groups, mainly involved in nutrient metabolisms, such as amino acid, vitamin, nucleotide and organic acid metabolism (refer to Supplementary files). We explored the roles of the gut microbiome in modulating erucic acid, spermidine, adenosine, and L-5-Hydroxytryptophan metabolism by mapping the differential enzymes involved in the production of these metabolites (refer to Supplementary files). We observed that adenosine deaminase involved in adenosine metabolism to inosine [9] was up-regulated in ISS. There was a higher abundance of 21 gut bacterial strains expressing adenosine deaminase in ISS children (Fig. 4A). In addition, we observed that arginine decarboxylase involved in the arginine and spermidine metabolism [10] was decreased in the ISS group relative to the control group. There was a lower abundance of 25 bacterial strains expressing arginine decarboxylase in ISS children (Fig. 4B). Furthermore, we found that the abundance of tryptophan decarboxylase was enriched in ISS relative to control subjects, which may result in increased L-5-Hydroxytryptophan metabolism to serotonin [11] (Fig. 4C). Unfortunately, we were unable to identify any strains harboring tryptophan decarboxylase.

### Multi-omics analysis

**Fig. 5:**
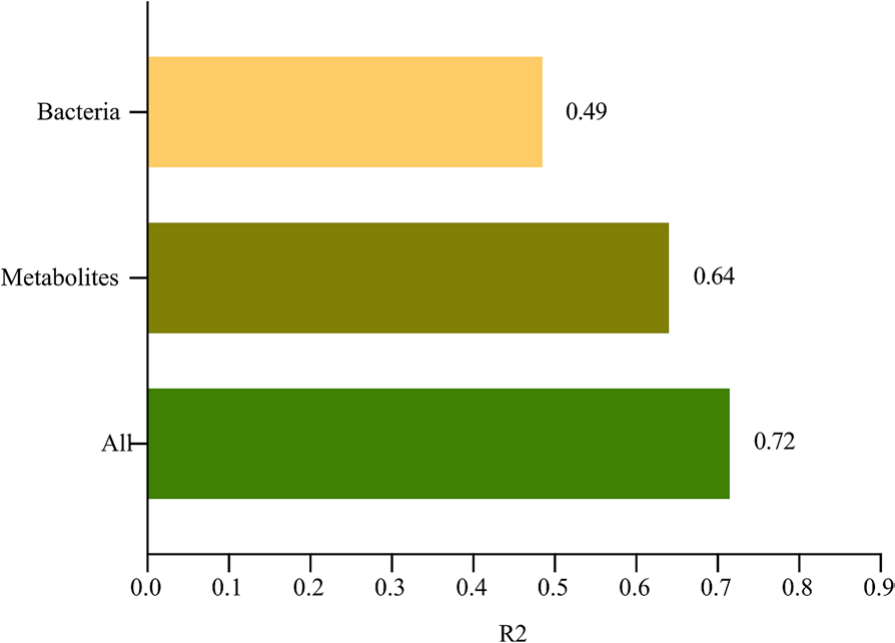
Inter-individual variation in clinical indicators explained by bacteria and metabolites

To compare the relative importance of fecal microbiome and metabolites in explaining the variability of inter-individual clinical indicators(age, height, weight, height SD and BMI), the proportion of variance explained by these two factors for the individual clinical indicators was calculated separately. We found that the type and abundance of gut bacteria and the composition of the metabolites could explain 49% and 64% of the variance, respectively (Fig. 5). Together, these two factors combined explained 72% of the variance in the clinical indicators.

**Fig. 6:**
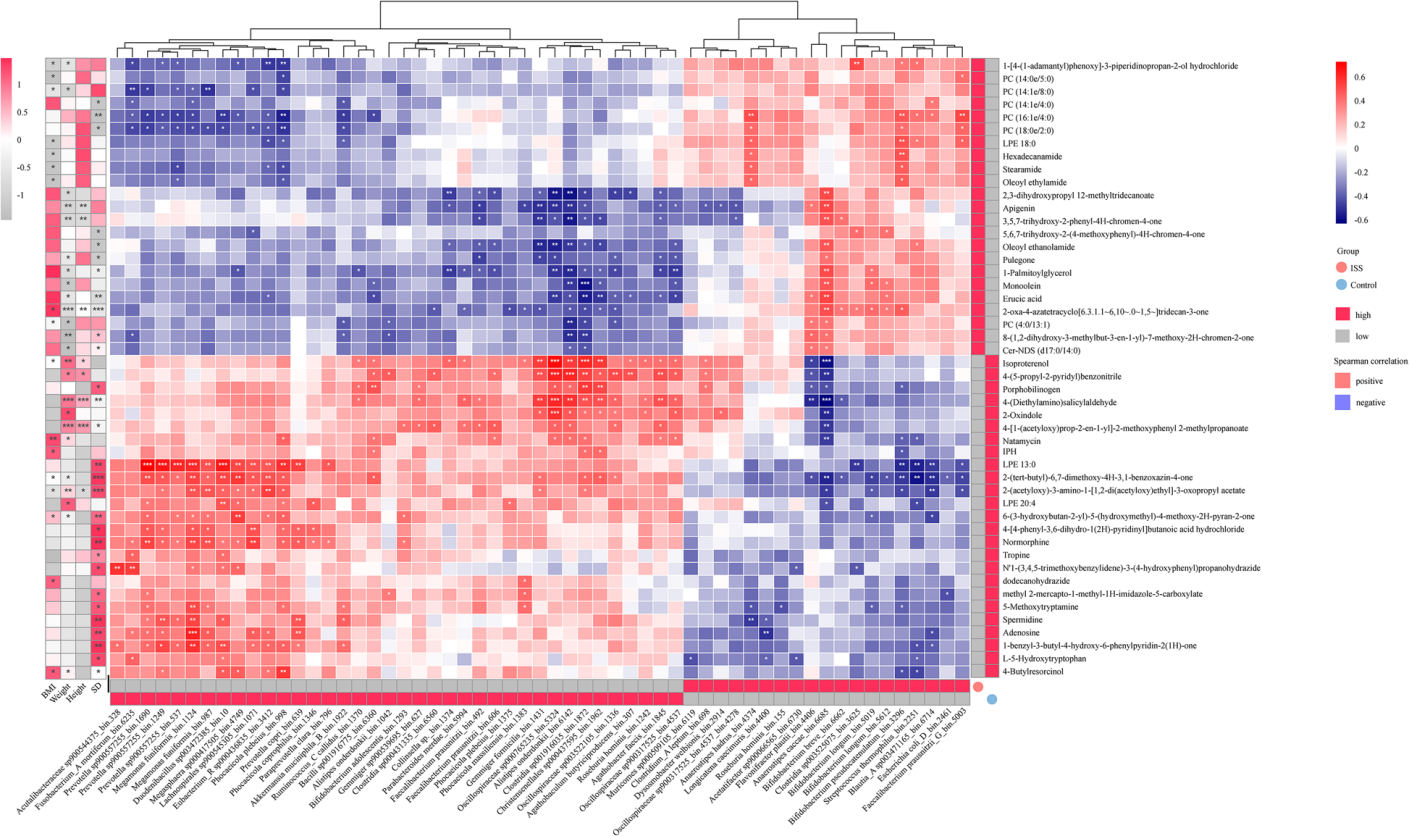
Spearman correlation analysis of clinical indicators, bacterial strains, and metabolities. * *p*-value < 0.05; ** *p*-value < 0.01; *** *p*-value < 0.001

We performed Spearman's correlation analysis to investigate whether the differential metabolites were correlated with the clinical parameters. There were 37 differential metabolites significantly associated with the height SD value (Fig. 6), of which 22 were positively correlated and 15 were negatively correlated. Spermidine, adenosine, and L-5-Hydroxytryptophan, enriched in the control group, were positively correlated with the height SD value. In addition, erucic acid was enriched in the ISS group and showed a significant association with the height SD value. We further assessed the association between the metagenomic abundance changes and clinical indicators by Spearman’s correlation analysis. There were 35 bacterial strains significantly associated with the height SD value (Fig. 6), of which 23 were positively correlated and 12 were negatively correlated. We observed *Longicatena caecimuris_bin.4400, Anaerostipes hadrus_bin.4374,* and *Streptococcus thermophilus_bin.2251* were negatively correlated with the height SD value and, *Christensenellales sp000437595_bin.1962* and *Bacilli sp001916775_bin.6360* were positively correlated with the height SD value. Moreover, we assessed associations between the metabolites and bacterial strains across all subjects. Given an FDR of 5%, 66 bacterial strains were correlated with 50 metabolites, presenting 461 significant associations (Fig. 6). Among them, we found *Longicatena caecimuris_bin.4400* was negatively correlated with adenosine*,* L-5-Hydroxytryptophan, and spermidine. *Anaerostipes hadrus_bin.4374* was negatively correlated with spermidine. *Streptococcus thermophilus_bin.2251* was negatively correlated with L-5-Hydroxytryptophan. *Christensenellales sp000437595_bin.1962* and *Bacilli sp001916775_bin.6360* were negatively correlated with erucic acid.

**Fig. 7:**
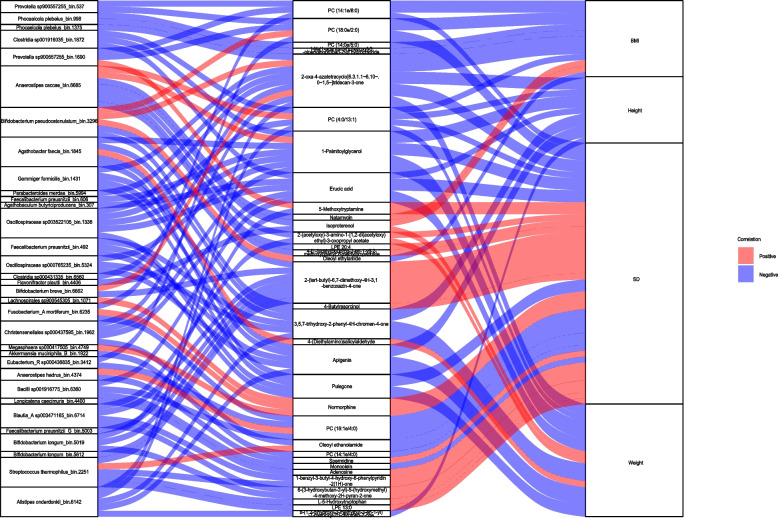
Sankey plots showed the correlation network between the gut microbiome and the clinical indicators was mediated by fecal metabolites. Red connections indicate positive correlations (FDR < 0.05), whereas blue connections indicate negative correlations (FDR < 0.05)

**Fig. 8:**
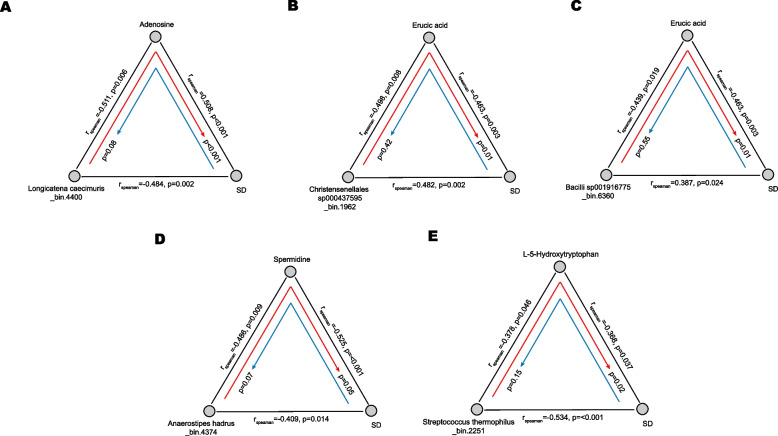
Mediation analysis and identification of interrelationships between the microbiome, metabolities, and the height SD. **A** Analysis of the effect of *Longicatena caecimuris_bin. 4400* on the height SD of children as mediated by adenosine. **B** Analysis of the effect of *Christensenellales sp000437595_bin.1962* on the height SD of children as mediated by erucic acid. **C** Analysis of the effect of *Bacilli sp001916775_bin.6360* on the height SD of children as mediated by erucic acid. **D** Analysis of the effect of *Anaerostipes hadrus_bin.4374* on the height SD of children as mediated by spermidine. **E** Analysis of the effect of *Streptococcus thermophilus_bin.2251* on the height SD of children as mediated by L-5-Hydroxytryptophan. The gray lines showed the Spearman associations between the two factors. Red and blue arrows indicate direct mediation and reverse mediation, respectively

A mediation analysis was also performed to investigate the links between clinical information, the microbiome, and metabolites. The analysis identified 126 mediation linkages (*p*_mediation_ ≤ 0.05, *p*_inverse-mediation_ ≥ 0.05) (Fig. 7) including 14 for the microbiome impact on the BMI through metabolites, 31 for the microbiome impact on the weight through metabolites, 50 for the microbiome impact on the height SD value through metabolites and 31 for the microbiome impact on the height through metabolites. We observed adenosine mediated a strong association of *Longicatena caecimuris_bin.4400* with the the height SD value (Fig. 8A). In addition, we found erucic acid could mediate the association of *Christensenellales sp000437595_bin.1962* and *Bacilli sp001916775_bin.6360* with the height SD value (Fig. 8B-C). Moreover, spermidine could mediate the association of *Anaerostipes hadrus_bin.4374* with the height SD value (Fig. 8D), and L-5-Hydroxytryptophan could mediate the association of *Streptococcus thermophilus_bin.2251* with the height SD value (Fig. 8E).

**Fig. 9:**
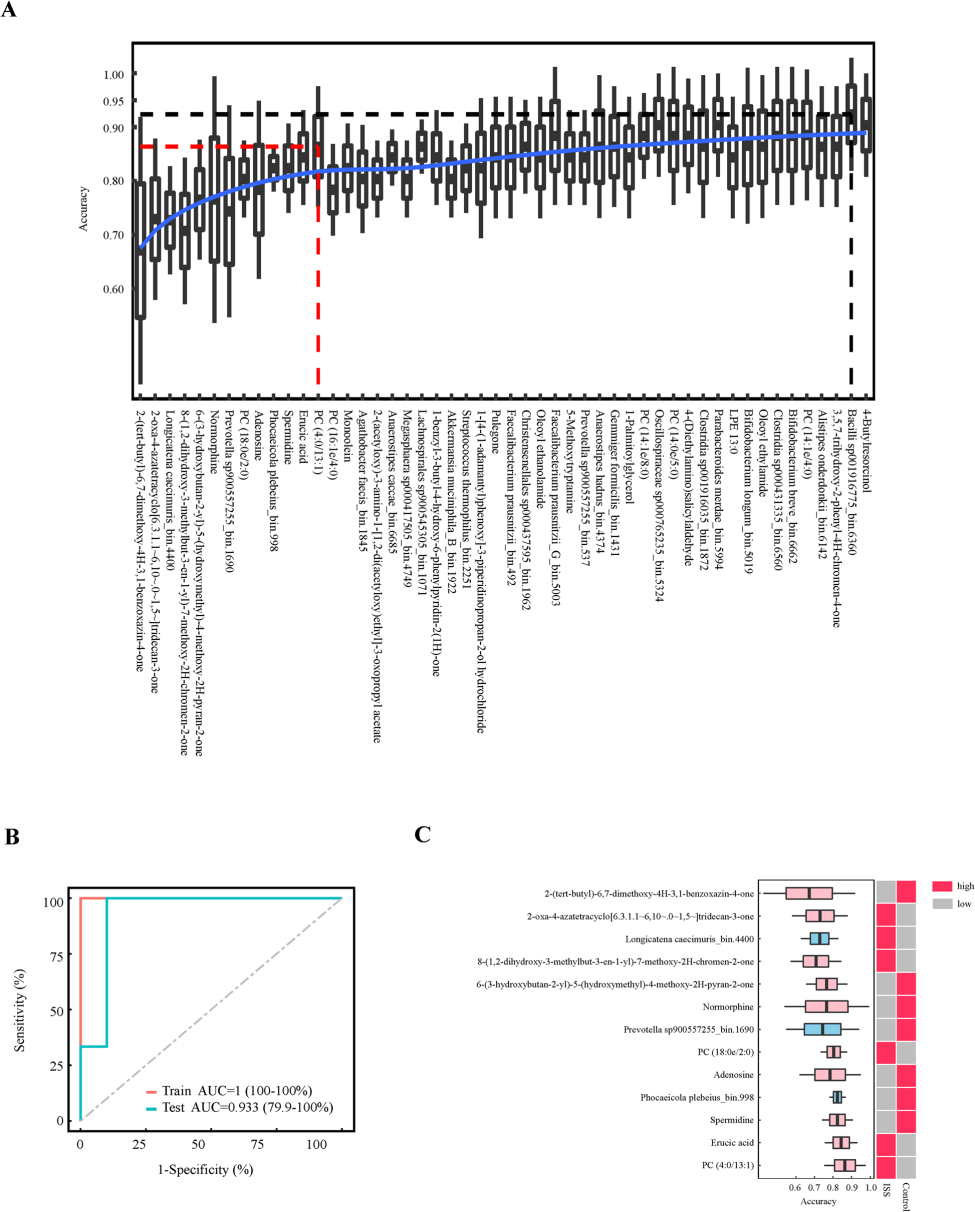
Metabolites and microbiome classifies ISS from Controls. **A** Recursive feature elimination (RFE) identified the 13 most important features. **B** ROC analysis was performed to evaluate these 13 features, and the AUC of the training set and test set were 100% and 93.3%, respectively. **C** The mean decrease in accuracy of 13 features was calculated. Red and blue shading denotes metabolites and microbiomes, respectively

Based on the results of mediation analysis, we included metabolites and bacterial strains of mediation linkages in the recursive feature elimination (RFE) model (Fig. 9A). Finally, 13 features were selected and used to perform receiver operating characteristic (ROC) analyses. The model had a significant discrimination for diagnostic accuracy [AUC(Test) = 0.933 (95%CI, 79.9–100%)] (Fig. 9B). Among the discriminatory features included in the classifier, 2-(tert-butyl)-6,7-dimethoxy-4H-3,1-benzoxazin-4-one had the greatest impact. The mean decrease in accuracy of 13 features was calculated in Fig. 9C.

## Discussion

In this study, using untargeted metabolomics and metagenomic sequencing, we identified significant differences in fecal metabolite profiles and intestinal microbiota in ISS children compared with healthy children. Our findings revealed that specific microbial taxa were significantly correlated with metabolites and the height SD value suggesting that the composition of the gut microbiota may influence children’s growth by regulating intestinal metabolic processes. In the clinic, we observe that some ISS children are thinner and shorter than healthy children, even though they have a balanced diet, adequate sleep and regular exercise. Through the correlation analysis between clinical indicators, we found the height SD value was positively related to BMI, supporting the notion that ISS children may have intestinal malabsorption.

Intestinal flora interacts with the human body by regulating metabolites. We identified 37 height SD-associated metabolites through untargeted metabolomics. Correlation analysis showed some fecal metabolites decreased in ISS, such as spermidine, adenosine, and L-5-Hydroxytryptophan, whereas erucic acid was enriched. Spermidine, as a polyamine, is known to play an important role in bone remodeling and fetal growth [12]. We also found decreased amounts of arginine in the gut of ISS children, a precursor for spermidine synthesis [13]. Adenosine is generated from the hydrolysis of adenine nucleotides and participates in energy metabolism. A study reported that chondrocytes could secrete adenosine to regulate the homeostasis and function of cartilage [14]. L-5-Hydroxytryptophan is produced from tryptophan, and further transformed into melatonin [11]. A study observed that L-5-Hydroxytryptophan could increase rapid eye movement sleep amount [15], suggesting that it may promote the secretion of growth hormone. Erucic acid is present in rapeseed oil and has been reported to inhibit animal growth [16]. A study showed that high erucic acid feeding decreased the absorption of nutrient substances, such as lipids and protein, inhibited growth, and disrupted the intestinal development of fish [16]. Among the differential metabolites, we observed that a number of lipids increased in ISS, while some amino acids and nucleotides decreased in ISS. KEGG pathway analysis showed that most metabolic pathways were enriched in amino acid and lipid metabolic pathways, indicating that ISS may have problems in the absorption and utilization of nutrients.

In this study, significant alterations in the abundance of gut microbiota were found in ISS children. The relative abundance percentages of butyrate-producing bacteria, such as *Faecalibacterium* and *Eubacterium* has been reported to be significantly reduced in ISS children [8]. However, our study revealed different butyrate-producing bacteria in the gut, *Butyricicoccus, Coprococcus*, *Fusobacterium_mortiferum*, *Alistipes_putredinis,* and *Coprococcus_comes,* were reduced in ISS children. About 95% of butyrate is absorbed and utilized within the colon, whereas only a small amount enters the circulation [17]. Butyrate plays a role in the regulation of the intestinal homeostasis and in repairing the intestinal barrier function [18]. We hypothesise that reduced amounts of butyrate in ISS children may have an influence on normal functions, such as digestion and absorption of nutrients. Moreover, we identified a total of 31 differential KEGG modules between the two groups. These differential modules were also mainly involved in the nutrient metabolism pathways such as amino acid metabolism and nucleotide metabolism. We speculate that disturbance of nutrient metabolism was of relevance to the gut ecosystem of ISS. Further, we explored the roles of the gut microbiota in modulating fecal metabolism by mapping the differential enzymes associated with the disturbed metabolisms. We found that the level of fecal spermidine and its relevant metabolic enzyme (arginine decarboxylase) were consistently decreased in ISS children. We also found a group of bacterial strains expressing arginine decarboxylase was also decreased in ISS children. In addition, the microbial metabolic enzyme (adenosine deaminase) involved in the metabolism of adenosine was up-regulated and a group of bacterial strains containing adenosine deaminase exhibited the same trend. Taken together, these findings suggest that the fecal levels of spermidine and adenosine in children with ISS may be collectively modulated by groups of gut bacterial strains. Bi-directional mediation analysis found 5 highly significant causal relationships. *Longicatena caecimuris_bin.4400* may affect children’s growth by regulating the metabolism of adenosine. Correlation analysis showed *Longicatena caecimuris_bin.4400* was negatively related to the height SD value and adenosine. We analyzed the enzymes expressed by the *Longicatena caecimuris_bin.4400* and found two enzymes (purine nucleoside phosphorylase and adenosine deaminase) involved in adenosine metabolism. Purine nucleoside phosphorylase catalyzes adenosine to the adenine [19] whereas adenosine deaminase catalyzes the hydrolytic deamination of adenosine to produce inosine [9]. The significantly enriched presence of *Longicatena caecimuris_bin.4400* in the gut of ISS children may thus play a role in the reduction of adenosine. The mechanism of how Christensenellales *sp000437595_bin.1962* and *Bacilli sp001916775_bin.6360* regulate the metabolism of erucic acid to affect children's growth remains to be further explored. A study reported that big broiler chicks had a higher abundance of *Christensenellales* compared to small chicks [20], which suggests that *Christensenellales* may promote growth. *Bacilli sp001916775_bin.6360* belongs to the class *Bacilli*. Several studies reported that a dietary supplementation with *Bacillus* strains help improved the growth performance of mice and broilers [21, 22].

A combination of 13 fecal metabolites and bacteria enabled construction of a disease classifier. The model discriminated ISS children from healthy children with a relatively high degree of accuracy (AUC of 0.953 in the test set). It is suggested that this identified metabolite signature has the potential of assisting clinical diagnosis and treatment in the future. In the clinic, we have observed that some ISS children are picky eaters. There is mounting evidence that dietary intake modulates the composition and function of the microbiome and metabolites [23]. In this study, we found erucic acid was increased in the ISS. Rapeseed and mustard oils have a high content of erucic acid [24], suggesting that a lower intake may be beneficial to ISS children. Further, based on the finding of low amounts of adenosine it may be desirable for ISS children to change to a diet with a high content of adenosine and arginine. From other clinical observations, some ISS children have insufficient sleep or poor sleep quality, and adequate sleep is important for normal growth and development. Since L-5-Hydroxytryptophan was also decreased in ISS children, and L-5-Hydroxytryptophan is mainly produced from tryptophan, supplementing their diet with food rich in tryptophan may also help to improve sleep quality. A study reported that children with stunting had lower serum concentrations of tryptophan [25]. Future research can explore whether children with ISS have lower serum tryptophan or deficits in conversion of tryptophan to L-5-Hydroxytryptophan.

Despite our efforts to correct batch effects using the Combat-seq method, this remains a limitation of our current study. Furthermore, the relatively small sample size constitutes another limitation. In our future research, we will collect data in a more systematic manner and expand the sample size in order to enhance the reliability of our findings. In conclusion, we identified gut microbiota and metabolite disturbances in children with ISS that may affect their growth and development. Through mediation analysis, we found several fecal metabolites closely related to the abundance of specific gut bacterial strains and found significant correlations with height SD value in ISS children. On this basis, our study points to dietary recommendations that may help promote normal growth and development of these children.

### Supplementary Information

Supplementary Material 1.

Supplementary Material 2.

## Data Availability

We uploaded the raw sequence data to the National Center for Biotechnology Information (NCBI) database (SUB12933851).
