# Supplementary material for: Gut microbiota and metabolic changes in children with idiopathic short stature
Source: BMC Pediatr. 2024 Jul 23;24:468. doi: 10.1186/s12887-024-04944-3 (PMC11265363; doi:10.1186/s12887-024-04944-3)
Supplement: Supplementary file 1 — Supplementary Material 1. [file 12887_2024_4944_MOESM1_ESM.docx]

Gut microbiota and metabolic changes in children with idiopathic short stature

Luyan Yan^1†^, Bin Ye^2†^，Min Yang^3†^，Yongsheng Shan^4†^，Dan Yan^1^，DanFeng Fang^2^，Kaichuang Zhang^1^，Yongguo Yu^1^*

^1^Department of Pediatric Endocrinology and Genetic Metabolism, Shanghai Institute for Pediatric Research, Xinhua Hospital Affiliated to Shanghai Jiao Tong University School of Medicine, Shanghai, China.

^2^Department of Pediatric Internal Medicine, Taizhou Central Hospital, Taizhou University Hospital, Taizhou, China.

^3^Department of Pediatrics, Shengjing Hospital of China Medical University, Shenyang, China.

^4^Department of Pediatrics, Xiaoshan Hospital Affiliated to Hangzhou Normal University, Hangzhou, China.

^†^These authors have contributed equally to this work and share first authorship.

*** Correspondence:**Yongguo Yu
yuyongguo@shsmu.edu.cn.

ORCID:0000-0002-4472-0910

**Supplementary Figure**


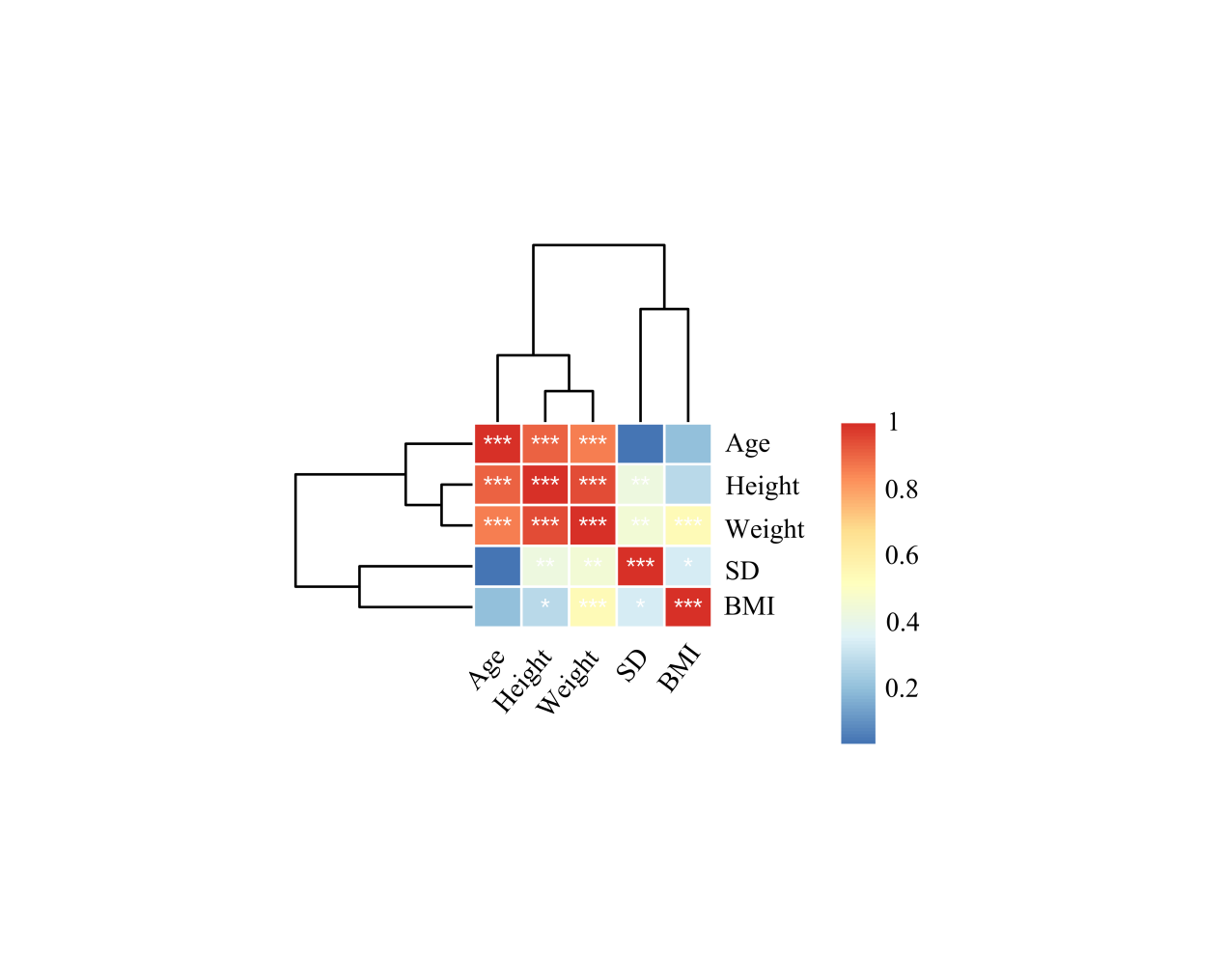


Fig 1. Spearman correlation analysis of clinical indicators. *p-value <0.05; **p-value<0.01；***p-value<0.001.


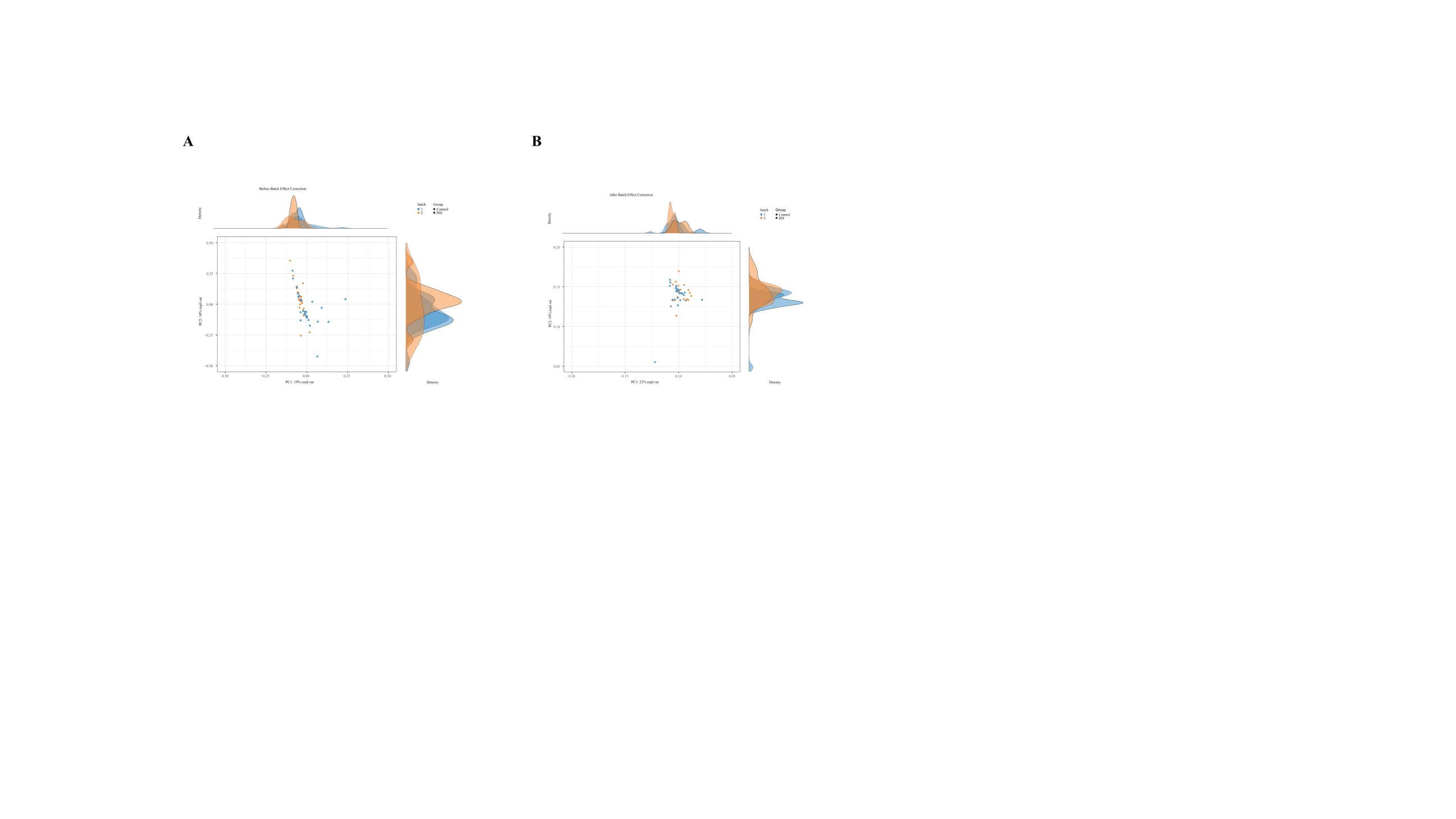


Fig 2. Batch effect correlation of microbiome data.


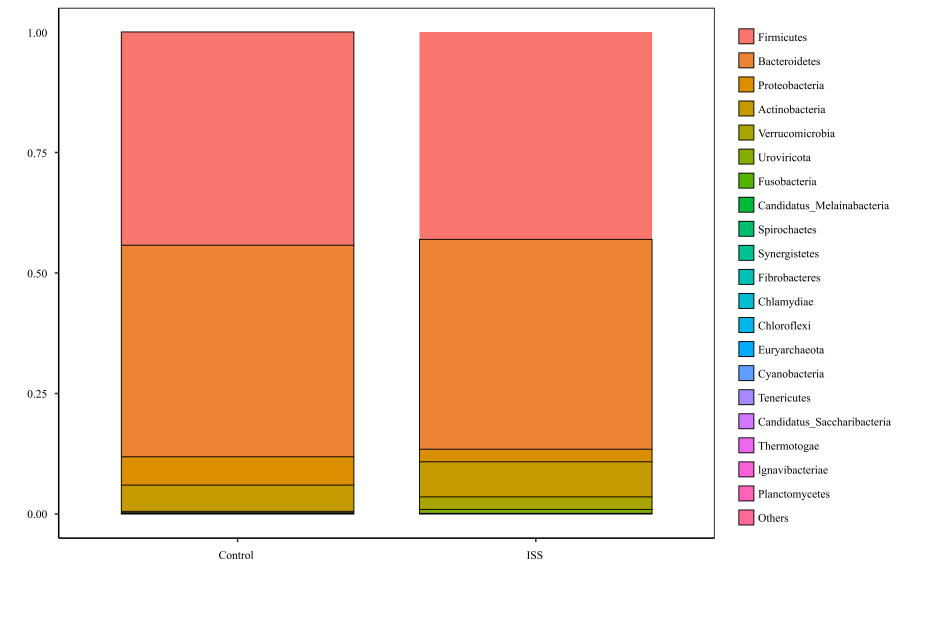


Fig 3. Species distribution at the phylum level.


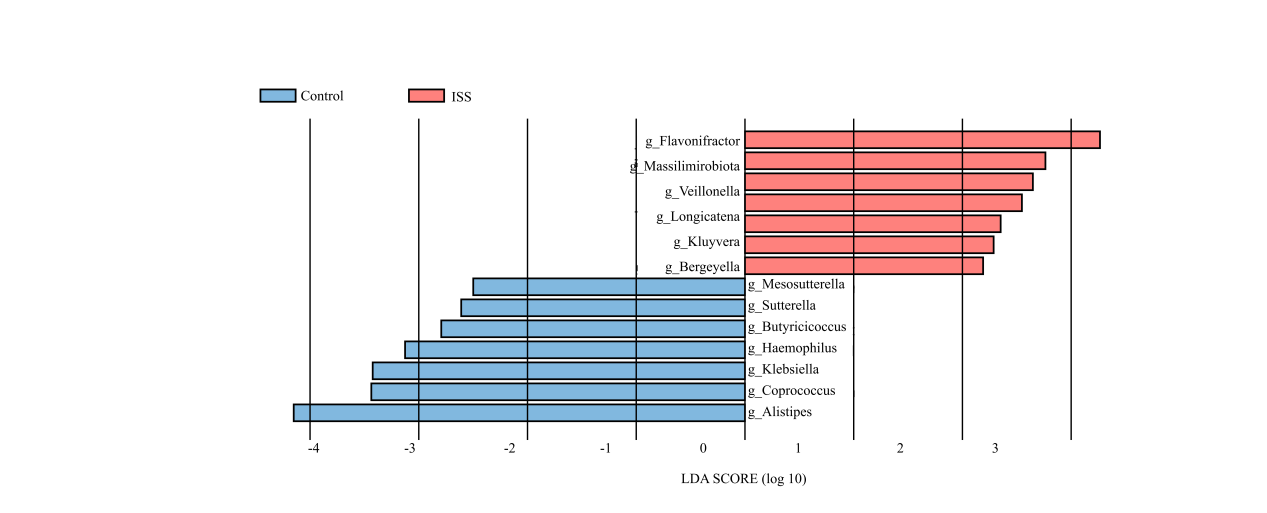


Fig 4. Microbiota with genus level difference.


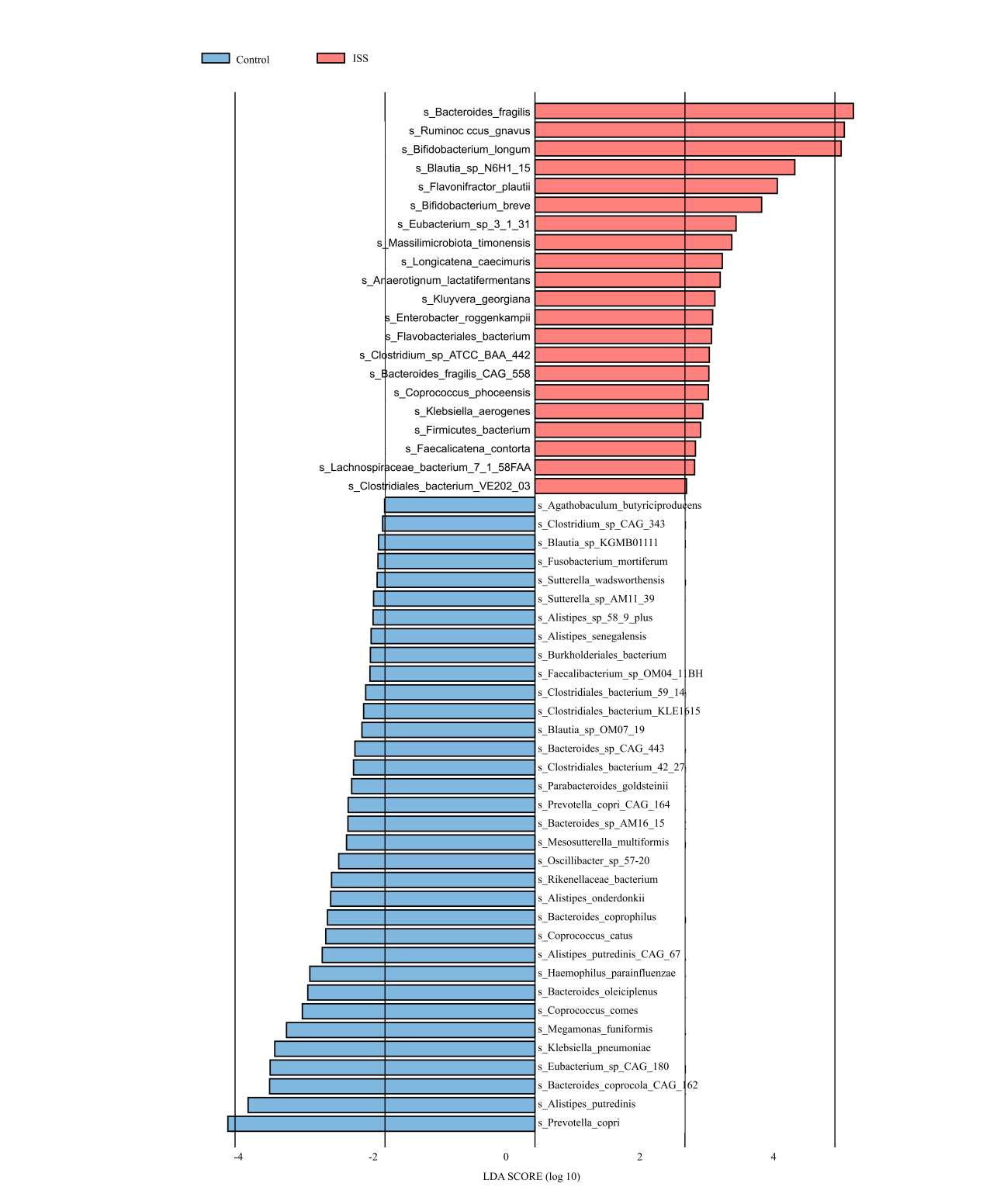


Fig 5. Microbiota with species level difference.


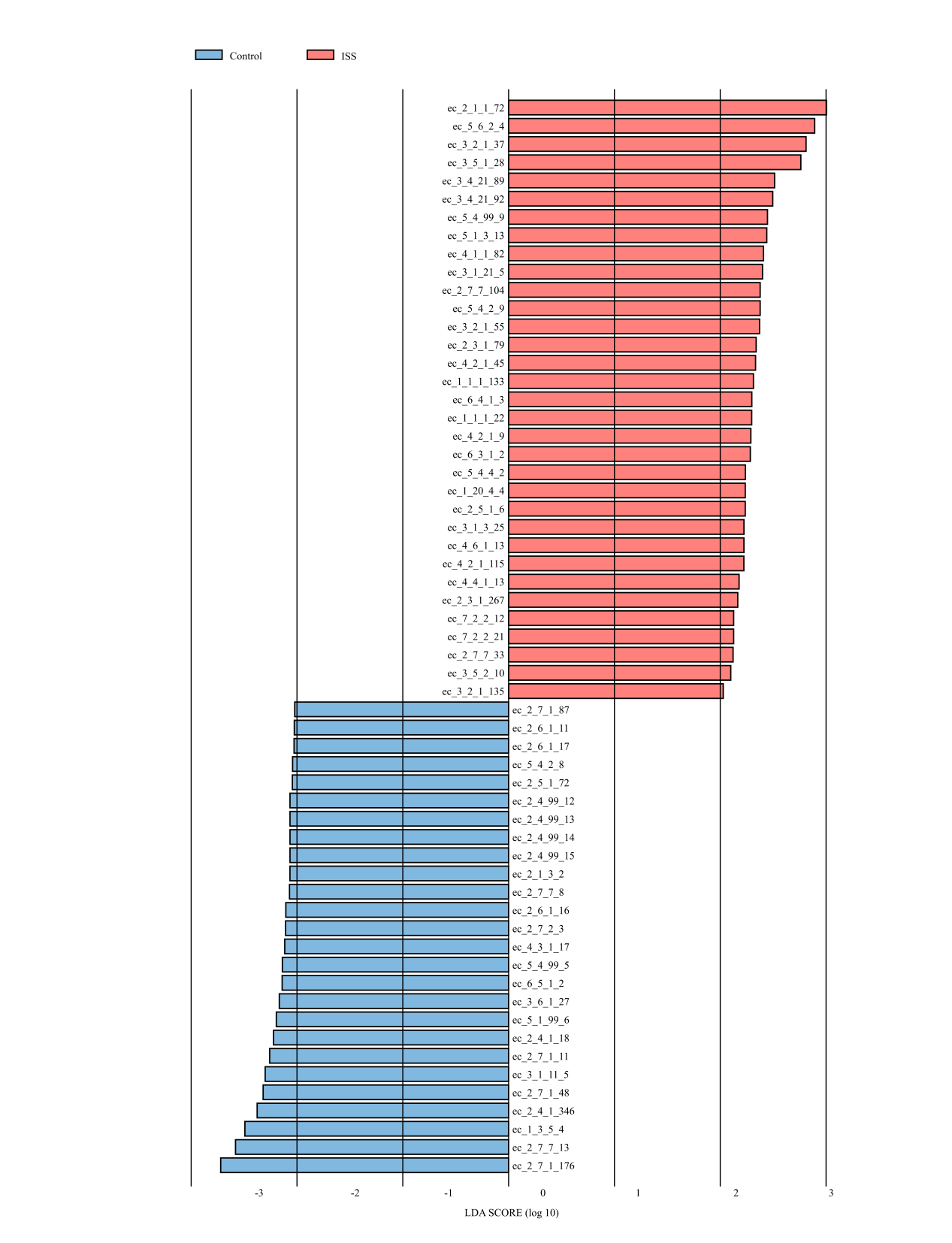


Fig 6. Linear discriminant analysis effect size (LEfSe) analysis displayed the differences in enzymes between the two groups.


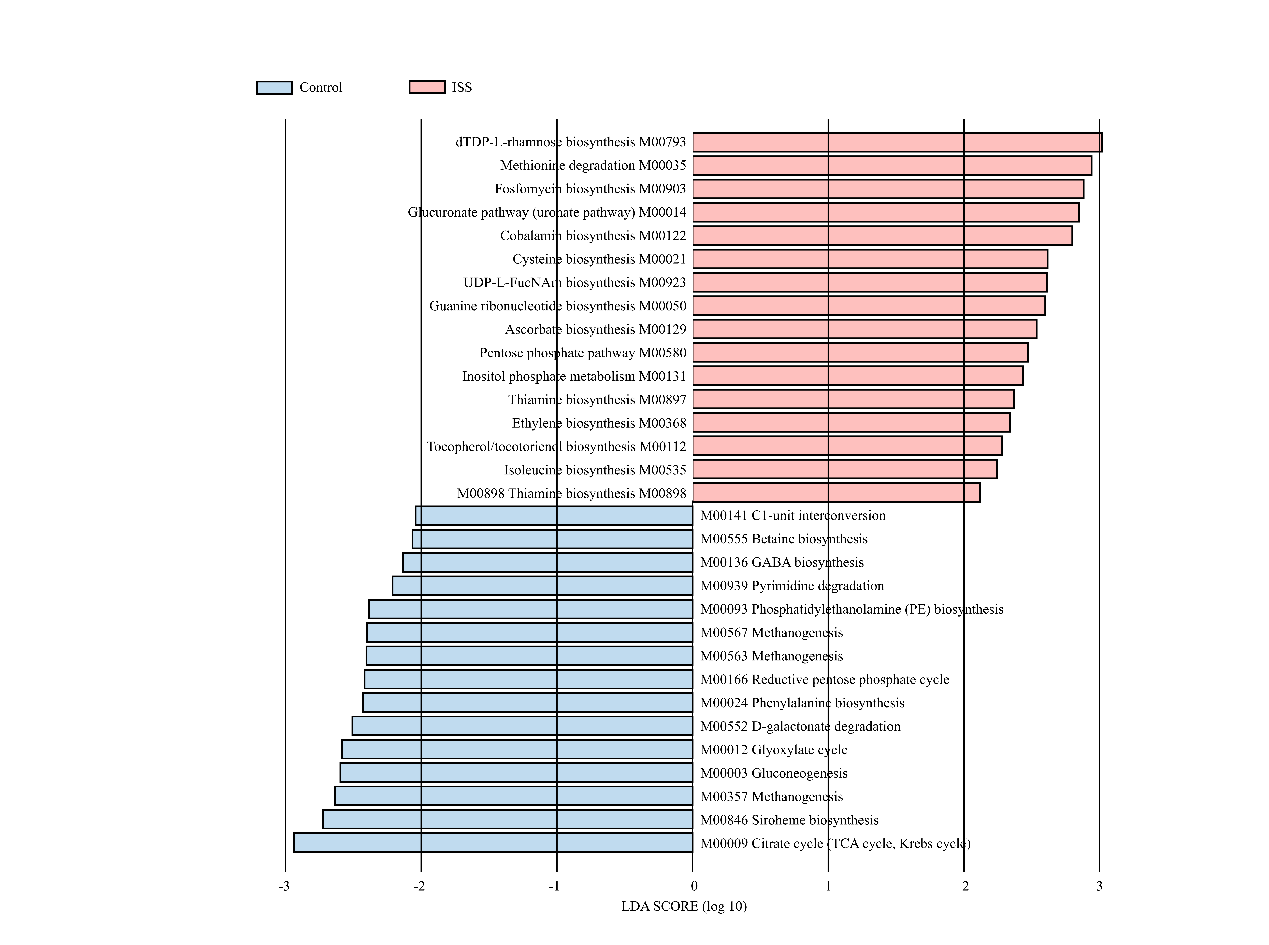


Fig 7. Linear discriminant analysis effect size (LEfSe) analysis displayed the differences in KEGG modules between the two groups.


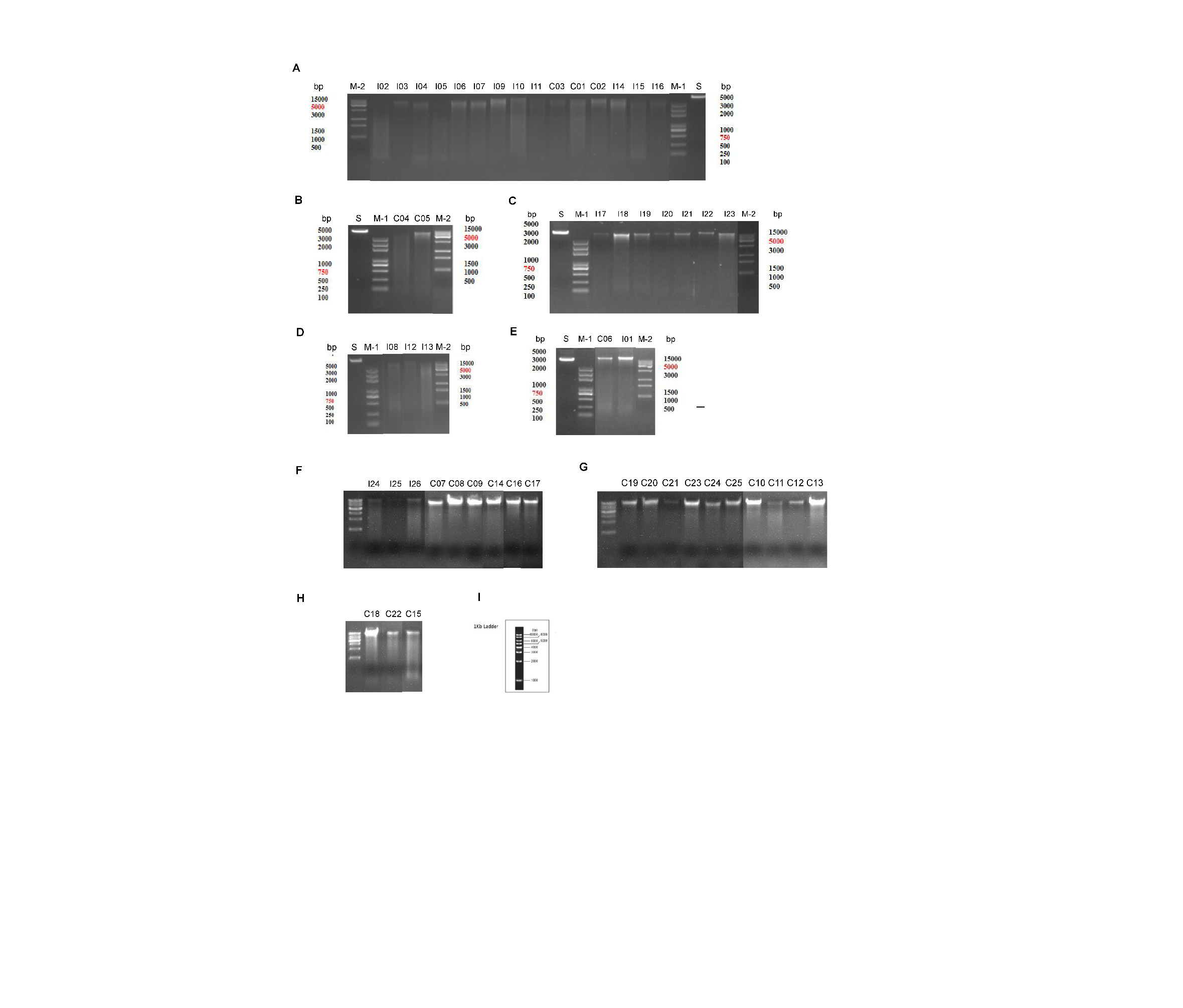


Fig 8. Gel electrophoresis of DNA from the fecal samples.(A-E)Gel electrophoresis of DNA from fecal samples of Batch 1.(F-H)Gel electrophoresis of DNA from fecal samples of Batch 2. (I)Ladder Band Pattern of DNA Gel Electrophoresis from batch 2.
